# Association between thyroid dysfunction during pregnancy and neonatal neurodevelopmental outcomes

**DOI:** 10.3389/fped.2026.1742074

**Published:** 2026-02-19

**Authors:** Yajun Kong, Xiaogang An

**Affiliations:** 1 Obstetrics Department, Shanxi Province Fenyang Hospital, Fenyang, Shanxi, China; 2 Pediatric Department, Shanxi Province Fenyang Hospital, Fenyang, Shanxi, China

**Keywords:** impact analysis, independent risk factors, neonatal neurodevelopment, pregnancy, thyroid dysfunction

## Abstract

This study aims to explore the impact of thyroid dysfunction (TD) during pregnancy on the neurodevelopmental outcomes of newborns. Participants were assigned to a thyroid dysfunction group (TDG, *n* = 92) or a normal control group (NCG, *n* = 150). Newborns underwent neonatal behavioral neurological assessment (NBNA), and serum thyroid hormone levels, including thyroid-stimulating hormone (TSH), free thyroxine (FT4), and free triiodothyronine (FT3), were measured. Within the TDG, subclinical hypothyroidism accounted for 43.48% (40/92), isolated hypothyroxinemia for 23.91% (22/92), subclinical hyperthyroidism for 17.39% (16/92), overt hypothyroidism for 9.78% (9/92), and overt hyperthyroidism for 5.43% (5/92). The total NBNA score, FT4, and FT3 levels were significantly lower in newborns from the TDG than those from the NCG (*P* < 0.001), whereas TSH levels were significantly higher (*P* < 0.05). Neurodevelopmental outcomes differed significantly among the various types of TD (*P* < 0.05). Correlation analysis showed that the total NBNA score of newborns in the TDG was negatively correlated with TSH level (r = −0.242, *P* < 0.05) and positively correlated with FT4 and FT3 levels (r = 0.464, r = 0.383, respectively; both *P* < 0.05). These findings indicate that TD during pregnancy significantly affects the neurodevelopmental outcomes of neonates, with the magnitude of effect varying according to the specific type of thyroid abnormality.

## 1 Introduction

Thyroid hormone (TH) plays a crucial role in fetal neurodevelopment and is involved in key processes such as neuronal proliferation, differentiation, migration, and myelination (1). Pregnancy represents a critical period for fetal neurodevelopment, particularly during early gestation, when the fetus depends entirely on maternal THs to support growth and neurological maturation (2). Epidemiological data indicate that the prevalence of thyroid dysfunction (TD) during pregnancy ranges from 2% to 17%, with subclinical hypothyroidism being the most frequent form, affecting approximately 2% to 5% of pregnant women (3). With the implementation of the two-child and three-child policies, the proportion of women of advanced maternal age has increased, thereby elevating the risk of TD during pregnancy.

In recent years, a growing body of evidence has demonstrated that TD during pregnancy is closely associated with adverse pregnancy outcomes, including miscarriage, preterm birth, gestational hypertension, and fetal growth restriction (4). Studies have found that children born to mothers with hypothyroidism during pregnancy show poorer intellectual performance, language development, and motor coordination, and these deficits may persist into school age and adulthood (5). Even mild forms of TD, such as subclinical hypothyroidism and isolated hypothyroxinemia, may exert detrimental effects on fetal neurodevelopment (6). However, the impact of TD on neonatal neurodevelopment during pregnancy remains controversial. Whether different types of TD exert distinct effects on neurodevelopment and whether the timing of treatment influences neonatal neurodevelopmental outcomes remains unclear. Although the American Thyroid Association recommends levothyroxine replacement therapy for the treatment of hypothyroidism during pregnancy, the optimal therapeutic window remains controversial (7). In addition, existing research (8) has largely focused on cognitive function in school-age children, while relatively few studies have examined the neurobehavioral development during the neonatal period. Neonatal behavioral neurological assessment (NBNA) is an important tool for evaluating neonatal neurodevelopment, allowing early identification of neurodevelopmental abnormalities and providing an opportunity for timely clinical intervention. Clinical evidence indicates that NBNA scores correlate with neurodevelopmental outcomes in infancy and early childhood, with lower scores being associated with an increased risk of subsequent motor and cognitive delays. Additionally, measurement of TH levels in newborns reflects intrauterine TH exposure and helps to assess the biological impact of maternal TD on the fetus.

Based on these research gaps and clinical needs, this study aims to explore the impact of TD during pregnancy on the neurodevelopmental outcomes of neonates, determine the risk levels associated with different types of TD, examine the influence of treatment timing on neurodevelopmental outcomes, and identify independent risk factors for adverse neonatal neurodevelopment. Using a retrospective cohort study design combined with NBNA assessment and neonatal TH level measurements, this study aims to (1) compare the effects of different types of gestational TD on neonatal NBNA scores, (2) examine the association between treatment timing and neonatal neurodevelopmental outcomes, and (3) identify independent risk factors for adverse neonatal neurodevelopment.

## 2 Materials and methods

### 2.1 Study participants

This study adopted a retrospective cohort design based on data extracted from the hospital information system. Pregnant women who delivered at our obstetrics department between January 2022 and June 2024 were included. The study was approved by the Ethics Committee of Shanxi Province Fenyang Hospital (Approval No. 2025082) and conducted in accordance with the principles of the Declaration of Helsinki. The requirement for informed consent was waived by the Ethics Committee of Shanxi Province Fenyang Hospital due to the retrospective nature of the study.

The inclusion criteria were: (1) singleton pregnancy; (2) registration at our hospital with antenatal care and complete thyroid function test data during pregnancy; and (3) delivery at our hospital with complete neonatal clinical data available.

The exclusion criteria were: (1) a history of thyroid disease or thyroid surgery; (2) TD diagnosed and treated before pregnancy; (3) comorbid endocrine disorders such as diabetes mellitus or adrenal disease; (4) previously diagnosed autoimmune diseases, including systemic lupus erythematosus, rheumatoid arthritis; (5) multiple pregnancy; (6) fetal chromosomal abnormalities or congenital malformations; (7) severe neonatal asphyxia at birth defined as an Apgar score ≤ 3 points at 1 min; and (8) incomplete clinical data.

**Figure 1:**
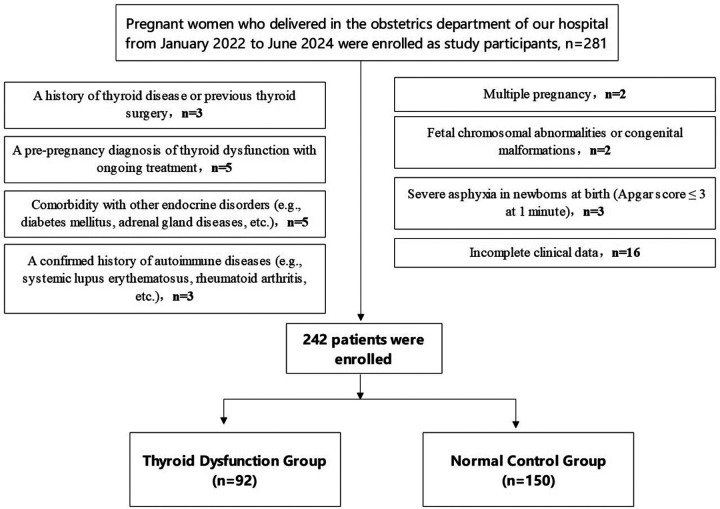
Flow diagram of participant screening.

Based on thyroid function test results during pregnancy, participants were assigned to the thyroid dysfunction group (TDG) (*n* = 92) or the normal control group (NCG) (*n* = 150). The participant flow diagram is shown in Figure 1.

### 2.2 Thyroid function testing and diagnostic criteria

All pregnant women underwent thyroid function testing in early pregnancy (8–12 weeks of gestation), including measurements of thyroid-stimulating hormone (TSH), free thyroxine (FT4), and free triiodothyronine (FT3). Analyses were performed using a chemiluminescent immunoassay (ADVIA Centaur XPT automated analyzer).

The diagnostic criteria were based on the 2017 American Thyroid Association (ATA) guidelines for the diagnosis and treatment of thyroid disease during pregnancy (9). Overt hypothyroidism was defined as a TSH level above the upper limit of pregnancy-specific reference value with FT4 below the lower limit. Subclinical hypothyroidism was defined as TSH above the upper limit of pregnancy-specific reference value, with FT4 within the normal range. Overt hyperthyroidism was defined as TSH below the lower limit of pregnancy-specific reference value with FT4 above the upper limit. Subclinical hyperthyroidism was defined as TSH below the lower limit of pregnancy-specific reference value, with FT4 within the normal range. Isolated hypothyroxinemia was defined as FT4 below the lower limit of pregnancy-specific reference value, with TSH levels within the normal range.

The pregnancy-specific reference ranges for thyroid function used in our hospital were as follows: during the first trimester, TSH 0.1–2.5 mIU/L and FT4 12.0–22.0 pmol/L; during the second trimester, TSH 0.2–3.0 mIU/L and FT4 10.3–21.5 pmol/L; and during the third trimester, TSH 0.3–3.0 mIU/L and FT4 8.4–19.8 pmol/L.

### 2.3 Treatment protocol

In principle, patients with TD initiated standardized treatment immediately after diagnosis. However, in routine clinical practice, the timing of treatment initiation varied because of delayed first prenatal visits, mild condition at initial diagnosis with subsequent progression, or delayed presentation for personal reasons. Patients with hypothyroidism were treated with levothyroxine sodium tablets. The initial dose was determined based on TSH levels: 25–50 μg/day for TSH 2.5–5.0 mIU/L, 50–75 μg/day for TSH 5.0–10.0 mIU/L, and 75–100 μg/day for TSH >10.0 mIU/L. Thyroid function was re-evaluated every four weeks, and the dose was adjusted to maintain TSH levels within the pregnancy-specific reference range. Patients with hyperthyroidism were administered propylthiouracil (PTU) at an initial dose of 100–150 mg/day, divided into 2–3 doses. For patients with subclinical hypothyroidism, levothyroxine was administered when serum TSH exceeded the upper limit of the pregnancy-specific reference range and thyroid peroxidase antibodies (TPOAb) were positive. Treatment was also provided when TPOAb was negative, but TSH levels exceeded 10 mIU/L. Current clinical guidelines do not provide definitive recommendations for the treatment of isolated hypothyroxinemia. In this study, some patients with isolated hypothyroxinemia received low-dose levothyroxine based on clinical assessment, whereas others did not receive treatment.

Based on the timing of treatment initiation, patients with TD were divided into an early treatment group (<12 weeks), a mid-term treatment group (12–24 weeks), a late treatment group (>24 weeks), and an untreated group.

### 2.4 Neonatal assessment

#### 2.4.1 Neonatal neurobehavioral assessment

All neonates were assessed by a trained neonatologist using the NBNA (10) between 3 and 7 days after birth. The NBNA consists of 20 items across five dimensions: (1) behavioral ability (6 items), (2) passive muscle tone (4 items), (3) active muscle tone (4 items), (4) primitive reflexes (3 items), and (5) general assessment (3 items). Each item is scored from 0 to 2, yielding a total score of 40. A total score <35 points was defined as abnormal neurodevelopment.

#### 2.4.2 Neonatal thyroid function testing

At 72 h after birth, heel blood samples were collected to measure TSH, FT4, and FT3 levels. The same analytic method used for maternal thyroid function assessment was applied. The reference ranges for neonatal thyroid function were as follows: TSH 0.7–15.2 mIU/L, FT4 11.5–28.3 pmol/L, and FT3 2.3–7.0 pmol/L (11).

#### 2.4.3 Neurodevelopmental assessment

Indicators of neurodevelopmental impairment, such as motor developmental delay (e.g., delayed milestones such as lifting the head and rolling over), language developmental delay (e.g., delayed vocalization and comprehension), and cognitive developmental abnormalities (e.g., impaired attention and memory), were recorded (12).

### 2.5 Data collection

Baseline characteristics of the two groups of pregnant women were collected, including (1) general information: maternal age, pre-pregnancy body mass index (BMI), primiparity, gestational hypertension, and gestational diabetes mellitus; (2) delivery-related information: gestational age at delivery, mode of delivery, and intrapartum complications; and (3) neonatal information, including sex, birth weight, length, and Apgar scores.

### 2.6 Quality control

(1) All physicians involved in NBNA received standardized training and were required to pass an evaluation test before performing assessments; (2) NBNA was conducted in a quiet, warm environment while the neonates were awake; (3) Laboratory testing were performed in strict accordance with standard operating procedures, with routine internal quality control and external quality assessment; (4) Data were independently double-entered to ensure accuracy; (5) All clinical data were collected and managed by designated personnel; (6) Cases were screened strictly according to the predefined inclusion and exclusion criteria, and only patients with complete clinical data were included in the final analysis.

### 2.7 Statistical methods

All statistical analyses were performed using SPSS version 26.0 (IBM Corp., Armonk, NY, USA). Continuous variables with a normal distribution were expressed as mean ± standard deviation (SD). Independent samples t-tests were used for comparisons between two groups, while one-way analysis of variance (ANOVA) was applied for comparisons among multiple groups, and the least significant difference (LSD-t) test was used for pairwise comparisons. For non-normally distributed data, variables were presented as median and interquartile range [M (Q1, Q3)], and group differences were assessed using the Mann–Whitney U test or Kruskal–Wallis H test. Categorical variables were presented as numbers and percentages [*n* (%)], and group comparisons were performed using the chi-square (*χ*^2^) test or Fisher's exact test. Spearman's correlation analysis was used to evaluate the correlation between TH levels and NBNA scores. Multivariable logistic regression analysis was conducted to identify independent risk factors for adverse neonatal neurodevelopmental outcomes. Variables with *P* < 0.1 in the univariable analysis were included in the multivariable analysis to avoid excluding potentially important variables due to overly stringent screening criteria. A *P*-value less than 0.05 was considered statistically significant.

## 3 Results

### 3.1 Comparison of baseline characteristics of the participants

Baseline clinical characteristics of the two groups of pregnant women, including maternal age, primiparity, pre-pregnancy BMI, gestational hypertension, and gestational diabetes, were compared. No statistically significant differences were observed between the two groups for these variables (*P* > 0.05) (Table 1).

**Table 1:** Comparison of baseline characteristics of the participants (mean ± SD)/ [*n* (%)].

Baseline characteristics	Thyroid dysfunction group (*n* = 92)	Normal control group (*n* = 150)	t/*χ*^2^	*P*
Average age (years)	29.26 ± 4.18	28.93 ± 3.82	0.530	0.629
Primipara (%)	59 (64.13)	99 (66.00)	0.088	0.767
Pre-pregnancy BMI (kg/m^2^)	22.78 ± 3.12	22.51 ± 2.89	0.494	0.684
Gestational hypertension (%)	14 (15.22)	17 (11.33)	0.770	0.381
Gestational diabetes (%)	17 (18.48)	24 (16.00)	0.249	0.618

### 3.2 Distribution of different types of TD

A total of 92 pregnant women with TD were included in this study. Among them, subclinical hypothyroidism accounted for the highest proportion (43.48%), followed by isolated hypothyroxinemia (23.91%), and subclinical hyperthyroidism (17.39%) (Table 2).

**Table 2:** Distribution of different types of thyroid dysfunction.

Types of thyroid dysfunction	Number of cases	Percentage (%)	Diagnostic gestational age (weeks)
Subclinical hypothyroidism	40	43.48	12 (8,16)
Isolated hypothyroxinemia	22	23.91	14 (10,18)
Subclinical hyperthyroidism	16	17.39	11 (8,15)
Overt hypothyroidism	9	9.78	10 (7,13)
Overt hyperthyroidism	5	5.43	9 (6,12)
Total	92	100.00	–

### 3.3 Comparison of NBNA scores between the TDG and the NCG

**Figure 2:**
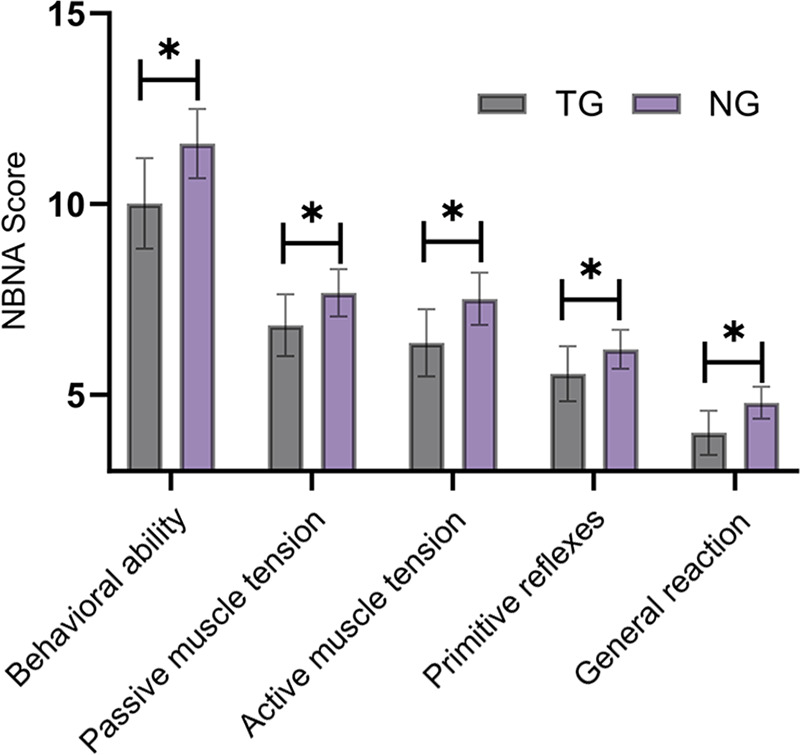
Comparison of NBNA scores between the thyroid dysfunction group and the normal control group. Scores of all NBNA dimensions were lower in the thyroid dysfunction group than those in the normal control group (*P* < 0.05). TG, thyroid dysfunction group; NG, normal control group. * indicates a statistically significant difference between the groups.

The total NBNA score of neonates in the TDG was 35.89 ± 2.82, which was significantly lower than the score of 38.15 ± 2.62 observed in the NCG (t = 6.327, *P* < 0.001). Scores across all NBNA dimensions were also significantly lower in the TDG than those in the NCG, with statistically significant differences between the groups (*P* < 0.05) (Figure 2).

### 3.4 Comparison of TH levels in neonates of different groups

**Figure 3:**
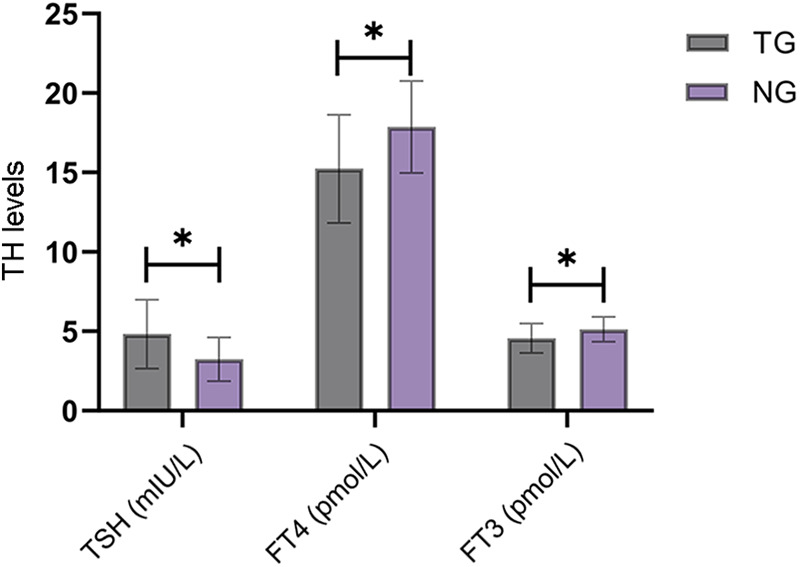
Comparison of TH levels in neonates between groups. TSH levels of the newborns in the thyroid dysfunction group were significantly higher than those in the normal control group, while FT4 and FT3 levels were significantly lower than those in the normal control group (*P* < 0.05). TG, thyroid dysfunction group; NG, normal control group. * indicates a statistically significant difference between groups.

Neonatal TH levels (TSH, FT4, and FT3) differed significantly between the TDG and NCG, with neonates in the TDG showing higher TSH levels [(4.82 ± 2.16) vs. (3.24 ± 1.38)] and lower levels of FT4 [(15.23 ± 3.41) vs. (17.86 ± 2.89)] and FT3 [(4.56 ± 0.92) vs. (5.12 ± 0.78)] compared with the NCG (*P* < 0.05) (Figure 3).

### 3.5 Comparison of dynamic changes in maternal TH levels during pregnancy between groups

**Figure 4:**
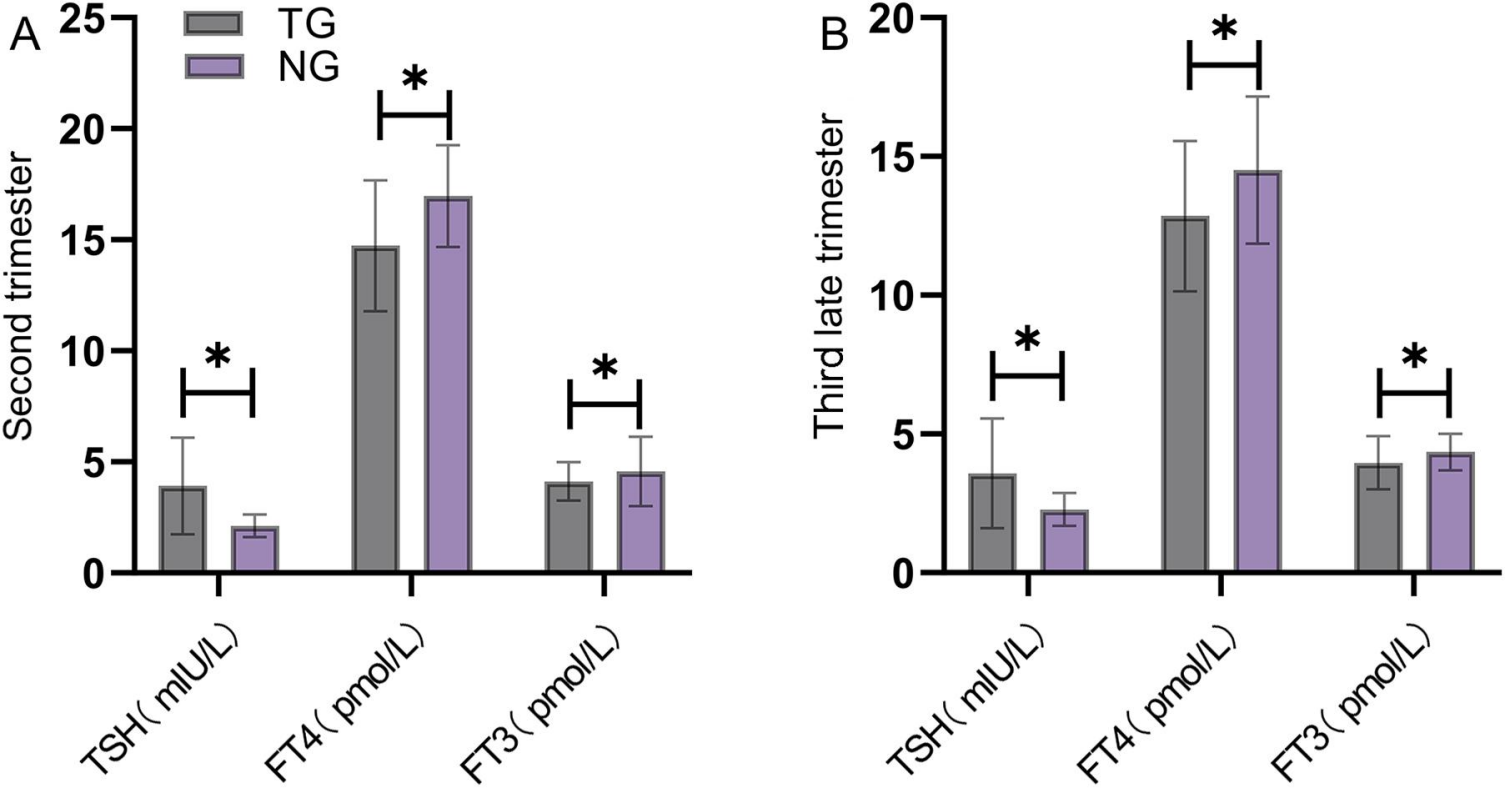
Comparison of dynamic changes in maternal thyroid hormone levels during pregnancy between groups. In both the second (Panel **A**) and third (Panel **B**) trimesters, TSH levels in the thyroid dysfunction group were significantly higher than those in the normal control group, while FT4 and FT3 levels were significantly lower than those in the normal control group (*P* < 0.05). TG, thyroid dysfunction group; NG, normal control group. * indicates a statistically significant difference between groups.

Maternal TH levels were collected and compared between the groups across pregnancy. In both the second and third trimesters, TSH levels in the TDG were significantly higher than those in the NCG, whereas FT4 and FT3 levels were significantly lower (*P* < 0.05) (Figure 4).

### 3.6 Analysis of the impact of different types of maternal TD on neonatal neurodevelopment

The impact of different types of maternal TD on neonatal neurodevelopment was evaluated. The results indicated that different types of TD had varying degrees of adverse effects on neonatal neurodevelopment, with differences in severity among the groups. The proportion of neonates with NBNA scores <35 was 30.00% (12/40) in the subclinical hypothyroidism group, 22.73% (5/22) in the isolated hypothyroxinemia group, and 18.75% (3/16) in the subclinical hyperthyroidism group. These proportions were significantly higher than those observed in the NCG (8.00%, 12/150; *P* < 0.001, *P* = 0.031, and *P* = 0.046, respectively). In the overt hyperthyroidism group, 20.00% (1/5) of neonates had NBNA scores <35, which did not differ significantly from that in the NCG (*P* > 0.05). The proportion of neonates with NBNA scores <35 in the subclinical hypothyroidism, isolated hypothyroxinemia, and subclinical hyperthyroidism groups was significantly higher than that in the NCG (8.00%, *P* < 0.05). Additionally, the rates of motor developmental delay, language developmental delay, and cognitive developmental abnormalities were significantly higher in the subclinical hypothyroidism, isolated hypothyroxinemia, and overt hypothyroidism groups compared with the NCG (*P* < 0.05) (Table 3). Notably, the sample sizes of the overt hyperthyroidism (*n* = 5) and overt hypothyroidism (*n* = 9) groups were relatively small, and these results should therefore be interpreted with caution.

**Table 3:** Analysis of the impact of different types of maternal thyroid dysfunction on neonatal neurodevelopment.

Group	*n*	NBNA <35 scores	Motor developmental delay	Language developmental delay	Cognitive developmental abnormalities
Normal control group	150	12 (8.00)	7 (4.67)	7 (4.67)	6 (4.00)
Subclinical hypothyroidism	40	12 (30.00)*	7 (17.50)*	6 (15.00)*	5 (12.50)*
Isolated hypothyroxinemia	22	5 (22.73)*	3 (13.64)*	3 (13.64)*	2 (9.09)
Subclinical hyperthyroidism	16	3 (18.75)*	2 (12.50)	1 (6.25)	1 (6.25)
Overt hypothyroidism	9	5 (55.56)*	3 (33.33)*	3 (33.33)*	2 (22.22)*
Overt hyperthyroidism	5	1 (20.00)	1 (20.00)	1 (20.00)	0 (0.00)

Compared with the normal control group.

* *P* < 0.05.

### 3.7 Correlation between TH levels and neonatal neurodevelopmental scores

**Figure 5:**
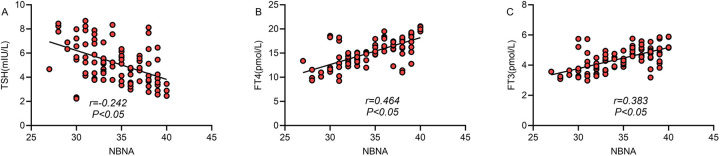
Correlation between thyroid hormone levels and neonatal neurodevelopmental scores. The total NBNA score of newborns in the thyroid dysfunction group was negatively correlated with TSH (panel **A**; r = −0.242, *P* < 0.05) and positively correlated with FT4 (panel **B**; r = 0.464, *P* < 0.05) and FT3 (panel **C**; r = 0.383, *P* < 0.05).

Spearman's correlation analysis was performed to assess the correlation between TH levels and NBNA scores in neonates born to mothers with TD. The total NBNA score was negatively correlated with TSH levels (r = −0.242, *P* < 0.05) and positively correlated with FT4 (r = 0.464, *P* < 0.05) and FT3 levels (r = 0.383, *P* < 0.05) (Figure 5).

### 3.8 Impact of treatment timing on neonatal neurodevelopmental outcomes

Subgroup analysis based on treatment timing showed a progressive decline in NBNA scores with increasing delay in treatment initiation. NBNA scores in the early-treatment, mid-treatment, late-treatment, and untreated groups were significantly lower than those in the NCG (*P* < 0.05). Furthermore, NBNA scores in the mid-treatment, late treatment, and untreated groups were significantly lower than those in the early-treatment group (*P* < 0.05). Regarding the incidence of neurodevelopmental abnormalities, the mid-treatment, late-treatment, and untreated groups exhibited higher rates compared with the NCG (*P* < 0.05). In addition, the late-treatment and untreated groups showed significantly higher rates than the early-treatment group (*P* < 0.05) (Table 4).

**Table 4:** Impact of treatment timing on neonatal neurodevelopmental outcomes.

Treatment timing	*n*	Total NBNA score	Rate of abnormal neurodevelopment
Normal control group	150	37.76 ± 2.13	12 (8.00)
Early treatment group (<12 weeks)	34	36.48 ± 2.41*	5 (14.71)
Mid-treatment group (12–24 weeks)	26	35.12 ± 2.68*,^#^	8 (30.77)*
Late treatment group (>24 weeks)	18	34.23 ± 2.87*,^#^	9 (50.00)*,^#^
Untreated group	14	33.79 ± 3.14*,^#^	8 (57.14)*,^#^

Compared to the normal control group.

* *P* < 0.05; compared to the early treatment group.

^#^ *P* < 0.05.

### 3.9 Multivariable logistic regression analysis of risk factors for adverse neonatal neurodevelopment

Multivariable logistic regression analysis was performed to identify independent risk factors for adverse neonatal neurodevelopment. Neonatal neurodevelopmental status (presence or absence of adverse neurodevelopment) was entered as the dependent variable, while other factors, such as overt hypothyroidism, subclinical hypothyroidism, and treatment timing, were included as independent variables. The analysis showed that hypothyroidism, delayed treatment timing, advanced maternal age, and preterm delivery were independent risk factors for adverse neurodevelopmental outcomes in neonates, with overt and subclinical hypothyroidism exerting the most significant effects (Table 5).

**Table 5:** Multivariable logistic regression analysis of risk factors for adverse neonatal neurodevelopment.

Variables	*B*	SE	Wald	OR (95%CI)	*P*
Overt hypothyroidism	2.131	0.382	31.142	8.423 (3.984–17.829)	<0.001
Subclinical hypothyroidism	1.537	0.268	32.885	4.651 (2.750–7.864)	<0.001
Isolated hypothyroxinemia	1.125	0.315	12.745	3.080 (1.661–5.711)	<0.001
Treatment timing (every 4-week delay)	0.642	0.186	11.917	1.901 (1.320–2.736)	0.001
Maternal age (every 5-year increase)	0.285	0.142	4.026	1.333 (1.007–1.755)	0.045
Preterm birth (<37 weeks)	0.893	0.297	9.041	2.442 (1.362–4.371)	0.003

## 4 Discussion

This retrospective cohort study evaluated the impact of maternal TD during pregnancy on neonatal neurodevelopmental outcomes. The findings demonstrated that TD was significantly associated with affected neonatal neurodevelopment, with the magnitude of effect varying across different types of dysfunctions. Early treatment was associated with improved prognosis. These results provide clinically relevant evidence to support individualized screening and treatment strategies for TD during pregnancy.

### 4.1 Epidemiological characteristics of TD during pregnancy

In this study, the overall incidence of TD during pregnancy was 38.02% (92/242), which was higher than that reported in previous studies (13). Several factors may explain this finding. First, the study participants consisted of pregnant women, which may have increased the detection of TD compared with the non-pregnant populations in other studies. Second, the region, diet, and environment may have affected the incidence rate. Third, the implementation of the two-child and three-child policies in China has increased the proportion of advanced maternal age. Previous studies (14) have demonstrated a significant association between advanced maternal age and TD during pregnancy. The results of this study also indicated that, with respect to the distribution of TD types, subclinical hypothyroidism was the most prevalent, followed by isolated hypothyroxinemia. This finding is consistent with that of previous studies and further suggests that subclinical hypothyroidism is a relatively common type of TD that requires sufficient attention during pregnancy.

### 4.2 Mechanisms of the impact of maternal TD on neonatal neurodevelopment

This study found that the total NBNA score of newborns in the TDG was significantly lower than that in the NCG, with impairments observed across all NBNA dimensions. Previous studies (15) have demonstrated that TH plays an important role in fetal neurodevelopment by promoting neuronal proliferation, differentiation, and migration, as well as regulating synapse formation and myelination. In addition to these established mechanisms, accumulating evidence indicates that THs may also be involved in the synthesis and release of fetal neurotransmitters and may modulate the fetal neurodevelopmental process by regulating the expression of genes involved in neurodevelopment (16). Based on these results, we propose that the fetal thyroid gland is not yet fully functional during early pregnancy (<12 weeks) and must therefore depend entirely on maternal THs. This period corresponds to a critical phase for the migration and differentiation of fetal cerebral cortical neurons. Maternal TD during this stage may adversely affect fetal neurodevelopment. Therefore, maternal thyroid function screening in early pregnancy is strongly recommended (17, 18). Although statistically significant differences in NBNA scores were observed between the TD groups and the NCG, the mean scores in both groups remained within the normal range (>35). As a result, the long-term clinical relevance of these differences requires confirmation through follow-up studies.

### 4.3 Differences in the impact of various types of TD

This study found that different types of TD have varying effects on neonatal neurodevelopment. Overt hypothyroidism was associated with the greatest degree of neurodevelopment impairment, followed by subclinical hypothyroidism and isolated hypothyroxinemia, reflecting a gradient related to the severity of TH deficiency. Previous studies (19) have shown that patients with overt hypothyroidism exhibit significantly elevated TSH levels and decreased FT4 levels, indicating profound thyroid hormone insufficiency. In contrast, patients with subclinical hypothyroidism exhibit elevated TSH levels despite normal FT4 levels, indicating decreased thyroid reserve. During early pregnancy, when fetus TH demand is high, maternal thyroid reserve may be insufficient to meet fetal requirements. The impact of isolated hypothyroxinemia on neonatal neurodevelopment remains controversial. However, the results of this study indicated that the rate of neurodevelopmental abnormalities in neonates in the isolated hypothyroxinemia group was higher than that in the NCG. This may reflect underlying maternal iodine deficiency or altered maternal peripheral TH metabolism. In contrast, the effects of hyperthyroidism on neurodevelopment are relatively mild, likely because moderate elevations in TH levels exert fewer negative effects on neurodevelopment than hormone deficiency (20, 21). Severe maternal hyperthyroidism may also indirectly affect fetal development by inducing placental insufficiency or preterm birth. Therefore, close clinical attention should be given to pregnant women with severe hyperthyroidism.

### 4.4 Clinical significance of neonatal TH levels

This study provided an in-depth analysis of neonatal TH levels and demonstrated that neonates in the TDG had elevated TSH levels and decreased FT4 and FT3 levels. These findings are clinically important because they reflect persistent effects of insufficient intrauterine TH exposure and suggest that maternal TD may affect the development and function of the fetal thyroid gland. Therefore, ongoing monitoring of thyroid hormone levels in neonates born to pregnant women with TD may be clinically warranted (22, 23). Correlation analysis further showed that the neonatal NBNA scores were negatively correlated with TSH (r = −0.242) and positively correlated with FT4 and FT3 (r = 0.464, r = 0.383), confirming the close relationship between TH levels and neonatal neurodevelopment and supporting the use of neonatal thyroid function testing as an auxiliary indicator for assessing neurodevelopmental risks.

### 4.5 Effect of treatment timing on neurodevelopmental outcomes

Another important finding of this study was that treatment timing had a significant impact on neonatal neurodevelopmental outcomes. The NBNA score and neurodevelopmental abnormality rate in the early treatment group (<12 weeks) were significantly more favorable than those in the mid-term treatment, late treatment, and untreated groups. Multivariable analysis showed that delayed treatment increased the risk of adverse neurodevelopment. This finding emphasizes the key role of initiating treatment during early pregnancy and is consistent with international guidelines (24). The first 12 weeks of gestation represent a critical period for fetal neurodevelopment. During this period, important processes such as neural tube closure, cerebral cortex formation, and neuronal migration take place. TH deficiency during this period may produce persistent adverse effects, whereas timely TH supplementation is associated with improved neurodevelopmental outcomes (25). In addition, this study showed that mid-term treatment also provided a protective effect compared with late treatment and no treatment, suggesting the presence of a treatment window during mid-pregnancy. This finding may be related to the different stages of brain development, as the second trimester is characterized primarily by neuronal differentiation and synapse formation.

### 4.6 Clinical significance of risk factor analysis

Multivariable logistic regression analysis identified several factors associated with adverse neurodevelopmental outcomes in neonates, with overt hypothyroidism showing the highest odds ratio, followed by subclinical hypothyroidism and isolated hypothyroxinemia. These findings suggest an association between the severity of TD and neonatal neurodevelopmental outcomes, consistent with previous reports. In addition, advanced maternal age was also found to be associated with adverse neurodevelopmental outcomes in neonates. Possible explanations for this association include a relatively reduced thyroid reserve in pregnant women of advanced age, a higher prevalence of autoimmune thyroid disease, and potentially placental insufficiency (26). These findings suggest that strengthening monitoring of thyroid function in pregnant women of advanced age may have clinical value. This study also found an association between preterm birth and adverse neurodevelopmental outcomes in neonates, which may reflect the loss of a critical period of intrauterine development and relative immaturity of nervous system development. It should be clarified that as an observational retrospective study, our analysis can only reveal statistical associations between these factors and neonatal neurodevelopmental outcomes, rather than establish causal relationships. Whether these associations are causal and what specific mechanisms are involved require further validation through prospective randomized controlled trials or other more rigorously designed studies.

### 4.7 Research innovations, limitations, and future directions

Through a detailed analysis of the impact of different types of TD and treatment timing on neonatal neurodevelopment, this study provides clinically relevant insights for the management of TD during pregnancy. The strengths of this study include the use of pregnancy-specific reference values for the diagnosis of TD, which improved the diagnostic accuracy. In addition, a standardized NBNA tool was used to evaluate neonatal neurodevelopment, enhancing the reliability of the results. However, several limitations should be acknowledged, such as potential selection bias inherent in the retrospective design, a relatively small sample size, and a short follow-up period. Furthermore, thyroid autoantibodies, including TPOAb and thyroglobulin antibodies, were not measured, which prevented assessment of the potential impact of autoimmune thyroiditis on neurodevelopmental outcomes. The exclusion of autoimmune disease relied primarily on previous diagnoses documented in medical records. In addition, the study population included only pregnant women who received regular prenatal care and delivered at a single hospital. This selection criterion may have excluded women with irregular prenatal care or poor socioeconomic conditions, potentially leading to an underestimation of the true impact of TD. Additionally, this study was unable to obtain data on important confounding factors, including iodine nutritional status, socioeconomic conditions, and maternal education level. Finally, due to the retrospective design, NBNA assessors were not blinded to maternal thyroid status; however, all assessments were conducted according to standardized procedures to minimize subjective bias. In light of these limitations, future research should focus on several key areas. First, large, multicenter prospective cohort studies are needed to verify the findings of this study. Second, longer follow-up periods should be incorporated to evaluate cognitive function and learning ability during school age. Third, optimization of different treatment plans, including initial dosing and dose adjustment strategies, should be further investigated. Finally, new neuroprotective strategies, such as antioxidant therapy, warrant exploration.

## 5 Conclusion

TD during pregnancy significantly affects neurodevelopmental outcomes of neonates, with the degree of influence varying across different types of TD. Hypothyroidism is associated with the most severe impact. Early diagnosis and timely treatment are associated with improved neonatal neurodevelopmental outcomes, whereas delayed treatment may increase the risk of adverse neurodevelopment. Clinically, strengthening thyroid function screening during pregnancy and implementing early intervention for high-risk pregnant women are essential to improve perinatal outcomes and promote prenatal and postnatal care.

## Data Availability

The original contributions presented in the study are included in the article/Supplementary Material, further inquiries can be directed to the corresponding author.
